# Elevated antistreptolysin O titer is closely related to cardiac mitral insufficiency in untreated patients with Takayasu arteritis

**DOI:** 10.1186/s12872-020-01364-w

**Published:** 2020-02-03

**Authors:** Lili Pan, Juan Du, Junming Zhu, Zhiyu Qiao, Yanlong Ren, Xinsheng Huang, Shichao Guo, Na Gao

**Affiliations:** 1 Department of Rheumatology and Immunology, Beijing Anzhen Hospital, Capital Medical University, 2 Anzhen Road, Chaoyang District, Beijing, China; 2 Department of Cardiovascular surgery, Beijing Anzhen Hospital, Capital Medical University, Beijing, China; 3 Department of Cardiology, Beijing Anzhen Hospital, Capital Medical University, Beijing, China

**Keywords:** Takayasu arteritis, Anti-streptolysin O, Mitral insufficiency

## Abstract

**Background:**

The etiology of Takayasu arteritis (TA) is unknown; however, a possible relationship between streptococcal infection and TA has been proposed. This study aimed to identify the clinical features and cardiac valvular involvement in untreated TA patients with an elevated antistreptolysin O (ASO) titer.

**Methods:**

In this retrospective study, the clinical characteristics and features of valvular involvement were compared in TA patients with or without an elevated ASO titer.

**Results:**

Of the 74 untreated TA patients, 13 patients were found have elevated ASO titers (17.6%). Mitral insufficiency was the most common in patients with elevated ASO (69.2%, 9/13), followed by aortic valve insufficiency (46.2%, 5/13) and tricuspid insufficiency (46.2%, 5/13), which were no significantly different than that in normal ASO group. The proportions of moderate to severe mitral (30.8% vs 1.6%, *p* = 0.000) and tricuspid valve (15.4% vs 1.64%, *p* = 0.023) insufficiency in the ASO positive group were significantly higher than those in the ASO negative group. The odds of mitral regurgitation in patients with elevated ASO titers were 3.9 times higher than those in the group with normal ASO titers (*p* = 0.053, OR = 3.929, 95% confidence interval [CI]: 0.983–15.694). Furthermore, the risk of moderate to severe mitral insufficiency in patients with elevated ASO titers was 41.6 times higher than that in patients with normal ASO titers (*p* = 0.002, OR = 41.600, 95% CI: 3.867–447.559).

**Conclusions:**

An increase in ASO titer is related to valvular involvement in TA and is closely linked to mitral insufficiency.

## Key-points

- Mitral insufficiency was common in TA patients with elevated ASO.
- There was a positive correlation between the titer of ASO and TA disease activity.
- Elevated ASO was risk factor to mitral involvement in untreated TA patients.

## Background

Takayasu arteritis (TA) is a type of chronic, systemic vessel vasculitis that mainly involves the aorta and its major branches, and is more common in young women of Asian descent [1]. Granulomatous inflammation in the media and adventitia is the pathological characteristic of TA, which results in fibrosis of large vessel walls, causing destruction of the elastic lamina and media [2]. Although the etiology of TA is not clear, several studies may have indicated a link with an antigen-driven, immune-mediated process [3, 4]. Streptococcal antigen is considered to play a important role in the autoimmune reaction of streptococcal infection-related disorders [5] and had been reported that TA sera reacted with streptococcal antigens [6]. The most widely used clinical antibody assays involve antistreptolysin O (ASO), which is valuable in patients with possible group A β-hemolytic streptococcal infections. Both TA and rheumatic fever (RF) could present with cardiac manifestations [7] and cause valvular disorders, resulting in systemic manifestations and making the diagnosis difficult [8]. Some reported cases of TA were diagnosed a few years after an initial diagnosis of RF [9, 10]. Cardiac involvement is the key determinant of morbidity and mortality in both TA and RF. They share similar clinical and epidemiological characteristics [11]. The incidence of cardiac valvular abnormalities in patients with TA is 7.0–36.7% [12]. Previous researchers have found a poor prognosis of TA with cardiac valvular involvement, which should be taken into consideration [13, 14].

Therefore, this study aimed to investigate the clinical features of cardiac valvular involvement in TA patients with positive ASO titers and to analyze the correlation between ASO titers and untreated TA disease activity.

## Methods

### Patients

This is a retrospective study, approved by Capital Medical University affiliated Beijing Anzhen Hospital Ethics Committee (approval number: 2018054X). Seventy-four consecutive untreated TA patients were enrolled, according to the criteria for classifying TA developed by the American College of Rheumatology in 1990 [15]. In total, 119 patients (74 were untreated) diagnosed with TA from January 2012 to December 2017 in Beijing Anzhen Hospital were recruited. We reviewed the records including demographic characteristics, clinical manifestations, laboratory tests, and imaging findings. Because acute RF affects cardiac valves, it was ruled out in the diagnosis of TA; therefore, there were no acute RF patients in our subjects. A modified version of Kerr’s criteria (Kerr score) [16] and the Indian Takayasu Clinical Activity Score (ITAS) [17] were used to assess TA disease activity. The differences in the clinical characteristics and features of valvular involvement were compared in patients with or without elevated ASO.

### Laboratory test

Four milliliters of venous blood were collected and the serum was isolated. An automatic biochemical analyzer (7600–120, Hitachi, Tokyo, Japan) was used to analyze the serum parameters. After the patients were diagnosed with TA, their serum was collected and tested by the hospital laboratory department, and the results were reported. The ASO titer was determined by immune transmission turbidimetry (DiaSys Diagnostic Systems, Shanghai, China) with ASO ≤ 200 IU/mL as negative and ASO > 200 IU/mL as positive. The erythrocyte sedimentation rate (ESR) was detected by the modified Westergren method.

### Angiographic features and echocardiogram

Magnetic resonance angiography, computed tomography angiography, and Doppler ultrasound were used to evaluate the thoracic and abdominal aorta and aortic branches. The lesions were classified according to the 1996 Numano classification [18]. Echocardiograms were obtained with a 5-MHZ ultrasound probe (EPIQ 7C, Philips, the Netherlands) to evaluate the cardiac structure and function following published guidelines [19–21]. The severity of mitral and tricuspid regurgitation was graded by using quantitative values similar to those for aortic regurgitation according to the American Society of Echocardiography recommendations [22].

### Statistical analysis

According to the normality, numerical data were expressed as quartiles or means ± standard error. And unpaired *t-* test or Mann-Whitney U test were used to determine the differences between TA patients with positive and negative ASO. For qualitative parameters, χ^2^ test was used assessed the differences between two groups. Variables including ASO, ESR, IgA, IgG, Kerr Score, complement 3 (C3), and ITAS were computed to a multivariate backward stepwise logistic regression analysis (*p* = 0.05 entry and *p* = 0.10 removal criteria) to predict the independent risk factors for the valvular involvement in TA patients. All *p*-values were two-tailed and *p* < 0.05 was interpreted as statistical significance. SPSS 16.0 statistical software (SPSS Inc., Chicago, IL, USA) was used for all the statistical analyses.

## Results

### General characteristic of untreated TA patients with positive ASO

There were 60 females (81.1%) and 14 males (18.9%). The median duraion of the disease was 48 (6.0, 144.0) months. Thirteen patients (17.6%) had a positive ASO titer (≥200 IU/ml), including 11 females (84.6%) and two males (15.4%). The average age of disease onset was 29.5 years (range 19.0–48.0) and the disease course ranged from 1 month to 3 years, with a median course of 6 months. The ASO titer ranged from 226 to 991 IU/ml. The most common angiographic type was Numano type V (30.8%, 4/13), followed by type I (23.1%, 3/13) and type IIb (23.1%, 3/13). In three patients (23.1%), the disease was complicated with pulmonary artery involvement and in one (7.7%), with coronary artery involvement (Table 1).

**Table 1:** General characteristics of untreated TA patients with elevated ASO titer

No	Disease duration (month)	Symptoms	Numano Type	Features of valvular involvement	ASO titer (IU/ml)
1	1	Left upper limb weakness	IIb	Mild mitral insufficiency	276
2	120	Dizziness	V, P+	Mild aortic insufficiency, mitral valve thickening, calcification and adhesion, moderate mitral stenosis and insufficiency	980
3	144	Dizziness, Fatigue	V	Mild aortic insufficiency, mitral valve calcification and prolapse, moderate mitral insufficiency, mild tricuspid insufficiency	398
4	5	Chest tightness	V	Mitral annulus dilatation and valve edge thickening, severe mitral insufficiency, moderate tricuspid insufficiency, pulmonary hypertension (Systolic pulmonary artery pressure: 70 mmHg)	580
5	12	Double upper limb weakness	I	Mild aortic insufficiency, mitral valve thickening, mild mitral insufficiency	709
6	6	Headache, fever, Fatigue	IV	None	560
7	1	Chest tightness, Palpitations	I	None	269
8	180	Chest tightness	V	Severe mitral insufficiency, mild tricuspid insufficiency, mild pulmonary insufficiency, pulmonary hypertension (Systolic pulmonary artery pressure: 50 mmHg)	226
9	1	Headache	I	Aortic valve edge thickening, mild mitral insufficiency, mild tricuspid insufficiency	991
10	48	Left upper limb weakness, Palpitations	IIa, P+	Aortic valve edge thickening, severe aortic insufficiency, mild mitral insufficiency, severe tricuspid insufficiency,	274
11	360	Fatigue	III	None	279
12	3	Chest tightness	IIb, P + C+	Aortic valve edge thickening, moderate aortic insufficiency, mild mitral insufficiency, mild tricuspid insufficiency, pulmonary hypertension (Systolic pulmonary artery pressure: 59 mmHg)	268
13	60	Double upper limb weakness	IIb	Mild aortic valve insufficiency	544

### Clinical features and angiographic manifestation in untreated TA patients with positive ASO

We compared the clinical manifestations of TA patients with positive or negative ASO. There were no significant differences in age, sex, duration of disease, and body mass index (BMI). There were also no significant differences in the clinical features and angiographic findings between the two groups. No significant difference in pulmonary artery and coronary artery involvement was observed, no atrial fibrillation was found in TA patients with valvular involvement (Table 2).

**Table 2:** Clinical and lab features of untreated TA patients with positive or negative ASO

	ASO positive *n* = 13	ASO negative *n* = 61	*P*-Value
Female, n(%)	11 (84.6)	49 (78.7)	0.720
Age of onset (year)	29.5 ± 10.0	33.5 ± 12.5	0.281
Disease duration (month)	6.0 (1.0, 102.0)	48.0 (12.0, 168.0)	0.081
BMI (kg/m^2^)	23.3 ± 3.5	22.8 ± 3.5	0.641
Arteriosclerosis, n(%)	3 (23.1)	30 (49.2)	0.086
Hypertension, n(%)	5 (38.5)	13 (21.3)	0.191
T2DM, n(%)	0 (0.0)	4 (6.6)	0.342
Smoker, n(%)	2 (15.4)	11 (18.0)	0.820
Heart failure, n(%)	4 (30.8)	9 (14.8)	0.168
AF, n(%)	0 (0.0)	0 (0.0)	–
Aneurysms, n(%)	3 (23.1)	13 (21.3)	0.966
Dizziness, n(%)	7 (53.9)	27 (44.3)	0.529
Headache, n(%)	2 (15.4)	11 (18.0)	0.820
Asymmetry in BP, n(%)	6 (46.2)	23 (37.7)	0.571
Pulseless, n(%)	4 (30.8)	13 (21.3)	0.462
Chest tightness, n(%)	5 (38.5)	17 (27.9)	0.448
Chest pain, n(%)	2 (15.4)	15 (24.6)	0.474
Palpitations, n(%)	3 (23.1)	4 (6.6)	0.065
Carotidynia, n(%)	1 (7.7)	4 (6.6)	0.882
Erythema nodosum, n(%)	1 (7.7)	1 (1.6)	0.222
Blurred vision, n(%)	0 (0.0)	4 (6.6)	0.342
Fever, n(%)	3 (23.1)	9 (14.8)	0.460
Fatigue, n(%)	4 (30.8)	14 (23.0)	0.551
Weight loss, n(%)	2 (15.4)	3 (4.9)	0.172
Numano Type			
I	3 (23.1)	16 (26.2)	0.086
IIa	1 (7.7)	3 (7.7)	0.688
IIb	3 (23.1)	9 (14.8)	0.460
III	1 (7.7)	4 (6.6)	0.882
IV	1 (7.7)	1 (1.6)	0.222
V	4 (30.8)	25 (41.0)	0.493
P+	3 (23.1)	7 (11.5)	0.267
C+	1 (7.7)	14 (23.0)	0.214

Note: *BMI* Body mass index; *T2DM* type 2 diabetes mellitus; *AF* atrial fibrillation; *P+* pulmonary artery involvement; *C+* coronary artery involvement

### Comparison of laboratory values and disease activity between untreated TA patients with positive or negative ASO titers

We compared the laboratory parameters and disease activity indexes of the two groups of patients. The results showed that the level of serum immunoglobulin (Ig) G in patients with positive ASO was significantly higher than that in patients with negative ASO (16.6 ± 4.1 vs 12.9 ± 3.7, *p* = 0.002). There was no significant difference in other parameters such as blood routine, liver function, kidney function, Ig A, IgM, complement 3 (C3), and complement 4 (C4). No significant difference in the disease activity index such as ESR, C-reactive protein (CRP), Kerr score and ITAS was found between the two groups (Table 3).

**Table 3:** Laboratory parameters and disease activity of untreated TA patients with positive or negative ASO

	ASO positive *n* = 13	ASO negative *n* = 61	*P*-Value
WBC(10^9^/L)	7.0 ± 1.5	7.3 ± 2.2	0.643
NE(10^9^/L)	4.7 ± 1.4	4.7 + 1.7	0.979
LY(10^9^/L)	1.9 ± 0.6	2.1 ± 0.8	0.391
RBC(10^12^/L)	4.51 ± 0.41	4.5 ± 0.5	0.812
Hb (g/L)	122.8 ± 21.9	124.2 ± 19.3	0.809
PLT(10^9^/L)	278.6 ± 107.2	265.4 ± 90.1	0.645
ALT (U/L)	13.7 ± 10.5	18.2 ± 14.3	0.289
Cr (μmol/l)	63.1 ± 12.8	61.2 ± 31.1	0.833
GLU (mmol/l)	5.0 ± 0.6	5.2 ± 1.1	0.612
HCY (μmol/l)	11.5 ± 3.5	13.9 ± 9.5	0.388
RF (IU/ml)	6.5 (4.0,10.6)	5.2 (3.1,11.7)	0.222
IL-6 (pg/ml)	16.2 (3.3, 29.4)	5.1 (2.3, 12.5)	0.157
TNF-α (pg/ml)	9.6 (5.8, 22.6)	20.3 (7.7, 48.7)	0.210
IgA (g/L)	3.2 ± 1.4	2.5 ± 1.3	0.086
IgG (g/L)	16.6 ± 4.1	12.9 ± 3.7	0.002
IgM (g/L)	1.5 ± 0.6	1.4 ± 1.0	0.823
IgE (g/L)	35.6 (14.3, 176.7)	17.0 (9.7, 87.3)	0.151
C3 (g/L)	1.3 ± 0.3	1.2 ± 0.2	0.188
C4 (g/L)	0.2 ± 0.1	0.2 ± 0.1	0.657
ESR (mm/1 h)	23.0 (9.0, 72.0)	18.0 (8.0, 39.0)	0.352
CRP (mg/L)	4.5 (1.8, 23.6)	2.2 (0.4, 21.5)	0.334
Kerr Score	2.5 ± 0.7	2.4 ± 0.7	0.484
ITAS	8.9 ± 6.0	8.2 ± 4.8	0.682

Note: *WBC* white blood cell; *LY* lymphocyte; *NE* neutrophil; *PLT* platelet; *RBC* red blood cell; *Hb* hemoglobin; *ALT* alanine aminotransferase; *Cr* creatinine; *GLU* Glucose; *HCY* homocysteine; *RF* rheumatoid factor; *IL* interleukin; *TNF* tumor necrosis factor; *Ig* immunoglobulin; *C3* complement 3; *C4* complement 4; *ESR* erythrocyte sedimentation rate; *CRP* C-reactive protein

We analyzed the correlation between ASO titer and the indexes of disease activity. The results showed that the titer of ASO was positively correlated with ESR (*r* = 0.291, *p* = 0.040), Kerr score (*r* = 0.286, *p* = 0.044), ITAS (*r* = 0.295, *p* = 0.037), IgG (*r* = 0.502, *p* = 0.000), and C3 (*r* = 0.285, *p* = 0.047). There was no correlation between ASO titer and CRP, IgA, IgM, and C4.

### Cardiac valvular involvement in untreated TA patients with positive ASO

Of the patients with positive ASO, 76.9% (10/13) had abnormal valvular findings. The proportion with abnormal valvular findings in patients with negative ASO was 65.6% (40/61), which was not significantly different. In each group, one case showed mitral stenosis and the others were valvular insufficiency. Mitral valve insufficiency was most common in patients with elevated ASO, accounting for 69.2% (9/13) (valve thickening in three patients, calcification in two, adhesion in one, and prolapse in one), which was not statistically significant compared with patients with normal ASO. Aortic valve insufficiency (46.2%, 5/13) and tricuspid valve insufficiency (46.2%, 5/13) were not significantly different between the two groups. One patient in each group had pulmonary valve insufficiency, one positive ASO patient showed stenosis complicated with insufficiency of the mitral valve, which was not significantly different between groups. The incidence of multiple valve involvement in the positive ASO group was 61.5% (8/13), which was higher than that in the negative ASO group (34.4%, 21/61), although there was no significant difference (*p* = 0.069). Single valve involvement was found in two cases (15.4%) in the ASO positive group and 19 cases (31.2%) in the ASO negative group, with no significant difference.

The proportion of moderate to severe mitral insufficiency in the ASO positive group was significantly higher than that in the ASO negative group (30.8% vs 1.6%, *p* = 0.000). There was no significant difference between the two groups in mild mitral valve insufficiency (38.5% vs 45.9%, *p* = 0.624). Two patients in the positive ASO group and one in the negative ASO group had moderate to severe tricuspid insufficiency; the difference was statistically significant (15.4% vs 1.6%, *p* = 0.023), there was no significant difference between the two groups in mild tricuspid insufficiency (30.8% vs 27.9%, *p* = 0.833). There was no significant difference in aortic insufficiency between the two groups (Table 4).

**Table 4:** Features of cardiac valvular involvement of untreated TA patients with positive or negative ASO

Valvular involvement	ASO positive *n* = 13	ASO negative *n* = 61	*P*-Value
Total, n(%)	10 (76.9)	40 (65.6)	0.427
Aortic Valve insufficiency, n(%)	6 (46.2)	24 (39.3)	0.650
Mild	4 (30.8)	18 (29.5)	0.828
Medium to severe	2 (15.4)	6 (9.8)	0.559
Mitral valve insufficiency, n(%)	9 (69.2)	29 (47.5)	0.155
Mild	5 (38.5)	28 (45.9)	0.624
Medium to severe	4 (30.8)	1 (1.6)	0.000
Mitral valve stenosis, n(%)	1 (7.7)	1 (1.6)	0.222
Tricuspid valve insufficiency, n(%)	6 (46.2)	16 (26.2)	0.154
Mild	4 (30.8)	17 (27.9)	0.833
Medium to severe	2 (15.4)	1 (1.6)	0.023
Pulmonary insufficiency (mild), n(%)	1 (7.7)	1 (1.6)	0.222
Multiple valve involvement, n(%)	8 (61.5)	21 (34.4)	0.069
Single valve involvement, n(%)	2 (15.4)	19 (31.2)	0.252

We further analyzed the features of transesophageal echocardiography between the two groups in TA patients with valvular lesions. We found that 50% of the patients with elevated ASO had valve thickening (5/10), including 3 patients with aortic valve and 2 patients with mitral valve, which were significantly higher than those in ASO negative group (10.0%, 4/40) (3 patients with aortic valve and 1 patient with mitral valve) (*p* = 0.003). The ejection fraction was significantly lower in ASO positive group than that of normal ASO group (54.7 ± 13.8% vs 62.4 ± 9.2%, *p* = 0.042) (Table 5).

**Table 5:** Features of transesophageal echocardiography between the two groups in TA patients with valve involvement

Parameters	ASO positive *n* = 10	ASO negative *n* = 40	*P*-Value
Valvular prolapse, n(%)	1 (10.0)	3 (7.5)	0.794
Valvular thickening, n(%)	5 (50.0)	4 (10.0)	0.003
Valvular contracture, n(%)	0 (0.0)	1 (2.5)	–
Left ventricular end-diastolic dimension, (mm)	52.7 ± 12.5	47.7 ± 5.9	0.059
Ejection fraction, (%)	54.7 ± 13.8	62.4 ± 9.2	0.042
Left atrium enlargement, n(%)	3 (30.0)	14 (35.0)	0.765
Left ventricular diastolic dysfunction, n(%)	2 (20.0)	19 (47.5)	0.115
Pulmonary hypertension, n(%)	3 (30.0)	3 (7.5)	0.050

### Analysis of the risk factors of mitral and tricuspid valvular insufficiency in untreated TA patients

We used logistic stepwise regression analysis to analyze the effects of ASO, ESR, IgA, IgG, Kerr score, ITAS, and C3 on mitral and tricuspid regurgitation. The results showed that the risk of mitral regurgitation in patients with elevated ASO titer was 3.9 times higher than that in patients with normal ASO (*p* = 0.053, OR = 3.929, 95% CI: 0.983–15.694). The risk of moderate to severe mitral regurgitation in patients with positive ASO was 41.6 times higher than that in patients with normal ASO (*p* = 0.002, OR = 41.600, 95% CI: 3.867–447.559).

## Discussion

This study compared the clinical manifestations, laboratory findings, and heart valve disease characteristics of untreated TA patients with or without elevated ASO. We report, for the first time, that the proportion of mitral insufficiency is closely related with elevated ASO in untreated TA patients. Serum IgG was significantly higher in the ASO positive group than in the ASO negative group. In patients with positive ASO, the titer of ASO was positively correlated with ESR, IgG, and C3, and was paralleled to the TA disease activity index Kerr Score and ITAS.

Although the etiology of TA is unknown, it has long been suggested that TA pathogenesis is related to infections. Pathogen-triggered autoimmune response has also been proposed; however, convincing proof to support an association between streptococcus infection and TA is still lacking [23]. Both TA and RF could cause valvular abnormalities and present systemic clinical manifestations. Similar to our study, the association between an elevated ASO titer and TA has been described previously, with 19% of patients exhibiting an ASO titer twice the upper limit of normal [24]. Gan-gahanumaiah et al. reported a case of a female TA patient aged 29 years with heart failure, with a past history of RF, whose echocardiography suggested mitral and aortic regurgitation, and pulmonary hypertension [25]. Another case reported an 11-year-old boy with a markedly elevated ASO titer, who was initially diagnosed with acute RF; however, after admission, aortic wall thickening and enhancement were found on magnetic resonance angiography images, leading to a diagnosis of TA [7].

**Fig. 1:**
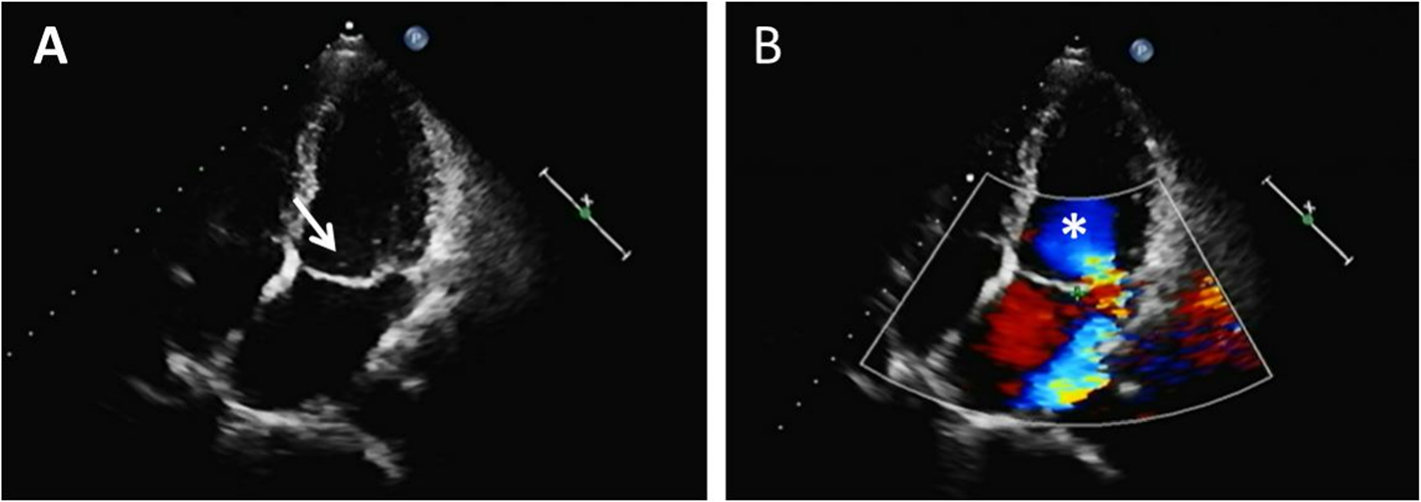
Echocardiography shows moderate mitral insufficiency in a 32-year-old TA patient with elevated ASO. Two - dimensional echocardiography suggested mitral valve calcification and prolapse (**a**), and Color Doppler ultrasound detected a large number of regurgitation signals in left atrium (**b**)

A previous study retrospectively assessed cardiac valvular structural abnormalities and dysfunctions and found that valvular involvement is common in TA patients, with the female to male ratio being nearly 5:1, similar to our result of 4.3:1 [26]. Aortic insufficiency was the most common finding in TA patients, followed by mitral insufficiency [12, 27, 28]. In this study, mitral insufficiency and aortic insufficiency were observed in 69.23 and 46.15% of TA patients with elevated ASO, respectively; therefore, mitral valve involvement was more likely in TA patients with elevated ASO. As shown in Fig. 1, a 32-year-old TA patient with elevated ASO, two - dimensional echocardiography suggested mitral valve calcification and prolapse, and Color Doppler ultrasound detected a large number of regurgitation signals in the left atrium. Moderate and severe mitral valve involvement was significantly more common in patients with ASO positive titers than in patients with normal ASO titers. Moderate and severe tricuspid insufficiency in TA patients with elevated ASO titers was also more frequent than that in those with normal ASO titers. Jing Li [12] reported that valvular abnormalities were found in 81.7% of TA patients, including single valve involvement in 67.2% and multiple valve involvement in 32.8%. In contrast, our study found that patients with elevated ASO, 61.5% of the TA patients exhibited multiple valvular lesions compared to 15.4% of patients with single valve involvement. These results suggest that streptococcus may aggravate valve damage in patients with TA. The remodeling of cardiac chambers may implicate the adjacent atrioventricular valves destroying the structure and function, resulting in dilatation of the valvular annulus and contortion of the subvalvular apparatus. This process may deteriorate heart failure, leading to severer manifestations and poorer prognosis [29], which is the main reason for mortality in TA patients [30]. According to our data, patients with valve problems may have lower EF, and that this would be a vicious circle between more severe valve problems and heart failure.

*Streptococcus pyogenes* activates both the innate and adaptive immune reactions. Multiple immune cells are involved including neutrophils, lymphocytes, monocytes, and macrophages. Several immune-mediated injury mechanisms are involved in valvular damage. Similar to the pathogenesis of rheumatic heart disease, peptides derived from cardiac endothelial cells in susceptible individuals resembled the epitopes of Streptococcus, and became self-antigens by this “molecular mimicry” phenomenon. The initial event was the recognition of self-antigens on antigen presenter cells, producing autoantibodies and autoreactive T cells that react against the patient’s self-antigens.

Autoantibodies activated endothelial cells, and promoted vascular cell adhesion molecule 1 expression. The following process was T-lymphocyte infiltration in the avascular valve matrix, resulting Th1-cytokine-mediated immune injuries. Furthermore, by epitope spreading, other valvular self-antigens including vimentin and collagen can be recognized and the immune response, amplified. Additionally, patients with group A streptococcal infections have elevated numbers of Th17 cells and higher serum IL-17 concentrations, indicating that Th17 responses occur in RF. These autoimmune responses were considered in association with TA [31, 32].

Recent studies have found that recombinant human α-enolase specific positive signals were detected in 57.1% of TA patients and specific streptococcal α-enolase positive signals were detected in 14.3%TA patients [6]. Streptococcal M protein binding to type IV collagen in the basement membrane can induce inflammation and fibrosis of valvular cusps [33]. Carbohydrate antigen (N-acetylβ-D-glucosamine) and Group A streptococcus M protein are believed to share the same epitope with human myosin and cardiac valve laminin; this structural similarity may result in antibody-mediated valve structure damage [34]. It was found that there was a large number of peripheral B-cells of active TA patients, and there were CD20+ B cells surrounding the granulomatous lesions [35]. Accordingly, it has been reported that the serum levels of antibodies that react with self-endothelial cells are elevated in TA patients. In vitro experiments verified that the autoantibodies induced endothelial cell proliferation through the mammalian target of rapamycin (mTOR) pathway [36]. We found that the serum IgG levels were markedly higher in TA patients with elevated ASO titers than in those with negative ASO titers and had a positive correlation with the ASO titers, suggesting that autoantibodies activated by the immune response after a streptococcal infection might play an key role in valve damage.

The main limitations were the retrospective nature of this study, because TA is a rare disease. In addition, our subjects were untreated patients, so the sample size was relatively small, and most of the patients are female. This study included all patients with valvular involvement because the cause of valvular insufficiency could not be fully determined; however, we did further divide the patients into mild and moderate to severe groups.

## Conclusions

In conclusion, we found that the proportion of mitral valve and tricuspid insufficiency in TA patients with elevated ASO titer was obviously higher than that in TA patients with normal ASO. ASO titer was positively correlated with IgG, ESR and C3 in TA patients with elevated ASO titer, which was parallel with the TA disease activity index. These results indicate that an elevated ASO is associated to valvular involvement in TA, which is closely related to mitral insufficiency. In TA patients with elevated ASO titer, cardiac valvular involvement should be monitored carefully.

## Data Availability

The datasets used and analysed during the current study are available from the corresponding author on reasonable request.

## Abbreviations

ASO: Antistreptolysin O
BMI: Body mass index
C3: Complement 3
C4: Complement 4
CRP: C-reactive protein
ESR: Erythrocyte sedimentation rate
Ig: Immunoglobulin
ITAS: Indian Takayasu clinical activity score
Kerr score: Modified version of Kerr’s criteria
mTOR: Mammal targets of rapamycin
RF: Rheumatic fever
TA: Takayasu arteritis
